# Listeria Cerebritis with Tumor Necrosis Factor Inhibition

**DOI:** 10.1155/2020/4901562

**Published:** 2020-04-24

**Authors:** Eileen Reilly, Justin Hwang

**Affiliations:** UW School of Medicine and Public Health, Madison, WI, USA

## Abstract

**Background:**

*Listeria monocytogenes* is historically a central nervous system pathogen of consideration in the very young, very old, and immune suppressed. Diagnosis of *Listeria* is based on positive bodily fluid culture or PCR testing. Cerebral edema is nonspecific and can be a manifestation of vasculitis, trauma, anoxia, ischemia, infarction, malignancy, or an infectious process. A main mechanism of immune protection against *Listeria* is tumor necrosis factor (TNF). Lenalidomide, an immunosuppressant, inhibits TNF. *Case Presentation.* A 61-year-old female with diabetes mellitus 2 and multiple myeloma treated with stem cell transplant and immunosuppressant (lenalidomide) was found to have cerebral edema after presenting with headache for 3 weeks and new focal neurologic deficits. Vitals signs were stable, with no meningeal exam findings and unremarkable initial serum testing. Blood cultures on days 0 and 2 of hospitalization as well as cerebral spinal fluid cultures were negative for infectious organisms. PCR testing of CSF was also negative for microorganisms. Brain biopsy was scheduled but postponed due to outstanding prion testing. The patient's focal neurologic deficits worsened prompting administration of dexamethasone after extensive negative infectious disease workup. By day 6, gross neurologic function deteriorated prompting transfer to higher level of care where the patient spiked a fever and one set of blood cultures revealed Gram-positive bacillus. Aggressive antimicrobial therapy was initiated, excluding ampicillin; however, this was later added. Blood culture further identified *Listeria monocytogenes*. By day 17, the patient suffered demise. Autopsy revealed brain microabscess lesions consistent with *Listeria*.

**Conclusion:**

Clinicians should employ prophylactic antimicrobial treatment for *Listeria* when caring for those patients presenting with cerebral edema who are immune suppressed with TNF inhibition no matter the initial exam findings, serum testing, and/or radiologic interpretation. If initial workup is negative and brain biopsy is needed to determine the next course of action in the patient with cerebral edema, transfer the patient to a higher level of care if unable to complete biopsy at your facility in an expedient fashion.

## 1. Introduction

A 61-year-old female with past medical history of multiple myeloma diagnosed July 2017 (stem cell transplant through Mayo Clinic Dec 2017 and chemotherapy instituted April 2018), type 2 non-insulin dependent DM (diagnosed Dec 2017 with last A1C of 11.2%- March 2019), unprovoked DVT in 2018 (Eliquis), and hypertension, who presented to the ER March 2019 with 2 day history of progressive weakness and decreased appetite. On day of admission, she was unwilling to get out of bed due to weakness, and her significant other noted right facial droop and right upper lid lag. In the emergency department, the patient reported blurred vision and headache for 3 weeks. She also reported no recent fever, vomiting, head injury, syncope, upper respiratory infection, abdominal pain, or urinary symptoms. With questioning, there had been no recent travel. The patient had suffered intermittent diarrhea that started after she was prescribed metformin. The patient was seen by her primary oncologist the day before, and serum testing was found to be baseline. She was compliant with her medications including metformin, Revlimid (lenalidomide), acyclovir, and Eliquis.

  Presenting vitals: BP 159/77 (patient position: lying); pulse 71; temperature 98.7°F (37.1°C); respiration 20; height 5′ 2″ (1.575 m); weight 154 lb 5 oz (70 kg); SpO_2_ 97%; BMI 28.22 kg/m^2^.
  Exam findings: the patient was alert and oriented X3 without distress and dismissive of her symptoms. No nuchal rigidity was found. Neurologic exam was completed by the neurology and the primary care team, which identified mild right facial asymmetry involving right lower facial muscle and mild right upper lid ptosis. Mild right arm weakness was 4/5 compared to 5/5 left arm but no drift. PERRLA and EOM were intact except chronic paralysis in the left lateral rectus muscle (congenital).
  Serum testing results: WBC 3.5 (4.1 day before); Hgb 12.3 (13.3 day before); Hct 39.2; MCV 89.5; MCH 30.3; MCHC 34.3; RDW 14.1; Plt 126 (135 day before); Neut % 56.3; lymph % 23.4; monocyte % 17.9; eosinophil % 0.9; Abs Neut 2.0; Abs lymph 0.8; Abs monocyte 0.6 (differential unchanged from previous day).
  BMP: potassium 2.9 (3.1 day before) and glucose 90. Anion gap was 19 but otherwise CMP normal.
  CPK: normal.

CT scan head per radiologist:

1. There was decreased attenuation throughout the white matter of the right cerebral hemisphere. This was asymmetric compared to the left. There was mild mass effect on the right lateral ventricle. Findings were worrisome for diffuse vasogenic edema. Differential would include sequela of prior therapy, underlying mass, less likely, given the asymmetry, and demyelinating process
2. Focal area with decreased attenuation in the right basal ganglia. Findings may represent a small lacunar infarction.
3. Mild diffuse cerebral volume loss.

**Figure 1:**
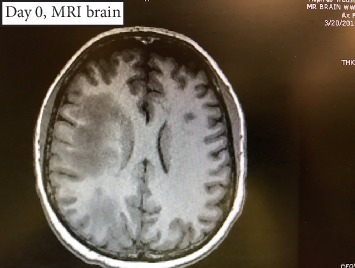
MRI head.

MRI interpretation by radiologist (Figure 1): Extensive patchy right frontoparietal and central midbrain perivascular (likely perivenular) enhancement. There is associated asymmetric white matter edema and midbrain edema. Differential considerations include vasculitis, intravascular lymphoma, amyloid angiopathy, granulomatous angiitis, and less likely demyelinating process. A small irregular area of restricted effusion in the right parietal lobe is nonspecific. Intravascular thrombus and venous infarction cannot be excluded.

The patient remained stable in the ER and was admitted to medical bed with close neurologic monitoring. Revlimid, metformin, and Eliquis were held. We continued prophylactic acyclovir.

Neurosurgery as well as Oncology was consulted with continued daily oversight by Neurology.

Neurosurgeon agreeable to brain biopsy if CSF analysis inconclusive since the concern was that her imaging finding suggestive of vasculitis or malignancy.

Day 1: patient stable with similar exam findings.
Day 1: lumbar puncture results.
    CSF analysis including flow cytometry: no malignant cells and cytology, and no monoclonal process.
    CSF fluid analysis: 83 lymphs, 11 monocytes, and 6 neutrophils.
    CSF cell count (2 bottles): RBCs 161/146 *μ*L; nucleated cells 32/20 *μ*L; neutrophils 2/6%, lymphs 80/83%, and monocytes 17/11%.
    Glucose 62 mg/dL and protein 69 mg/dL. Blood glucose was 95 at the time of the LP.
    CSF: complement factor elevated at 233, complement C3 elevated at 200, and complement C4 elevated at 48.
    CSF immunoglobulin testing unremarkable.
    CSF DNA testing: ACE, cryptococcus, *E. coli*, Epstein–Barr, herpes simplex, *Listeria monocytogenes*, *Neisseria meningitidis*, *Streptococcus* pneumococcus, GBS, CMV, Lyme, West Nile, neuron-specific enolase, VDRL, human herpes, varicella-zoster, human parechovirus, *H. influenza*: all negative.
*Initial CSF culture negative for growth after 6 days*
    JC virus and prion testing pending and not resulted until 1 week later but ultimately negative.
    Specific autoimmune and connective tissue disorder testing negative.
    Hepatitis C&B, HIV, and syphilis testing negative.
*Blood cultures obtained on day 1 to cover for rare bacterial pathogens were negative after 6 days*
    CT chest abdomen and pelvis on day 1: slight interval enlargement of left adrenal mass (present on previous imaging); otherwise no evidence of acute/new disease process.
    CTA head and neck on day 1: redemonstrated low attenuation diffuse right cerebral edema with possible small site of hemorrhage in the right frontal lobe. No vascular abnormality noted.
Day 2: Neurologic symptoms worsened with facial droop and right eye ptosis, and occlusion of vision. The patient reported increased weakness with limited use of the right arm for ADLs. The patient denied worsening headache, fever, or vomiting. Serum testing on this day revealed negative leukocytosis, however, ESR 37 and CRP 5.11.
Informal consultation completed with radiologist regarding a revisit on the differential diagnosis.
CSF cultures were still negative and given her negative CSF PCR testing for pathogens, and dexamethasone was initiated for cerebral edema and presumed cerebral vasculitis.
On the evening of day 2, the patient reported improvement of clinical symptoms with less ptosis and more strength.
Day 3: the patient with continual clinical improvement and requested discharge home in lieu of brain biopsy; however, this request was denied.
CT imaging unchanged and the patient afebrile.
The patient was scheduled for brain biopsy on Day 4 but was cancelled and rescheduled for Day 7 due to pending prion testing and lack of neurosurgical equipment backup while awaiting prion results. A further investigation regarding etiology of cerebritis was not further evaluated, given clinical improvement and stable vitals.
Day 6 in the AM: the patient's condition deteriorates with vomiting and escalating hypertension and tachycardia. Facial drooping on the right returned. The patient was afebrile in the AM on morning rounds with WBC 3.8, normal electrolytes, and unchanged renal function with glucose 230 and calcium 8.5. The patient was immediately transferred to ICU, and repeat imaging was ordered: CT head revealed no significant interval change in the cerebral white matter hypodensity with a small dense focus within the right frontal lobe similar to prior CT exam.
MRI head on day 6: Little change in abnormal brain MRI with diffuse parenchymal and perivascular enhancement in the right frontal lobe, right occipital lobe, floor of the 4th ventricle, and interpeduncular cistern. Minimal decrease in vasogenic edema. Small amounts of petechial blood products. Continue mild effacement of the right lateral ventricle and minimal right to left shift of midline. Differential: multifocal leukoencephalopathy, lymphoma, encephalitis, vasculitis, or myelomaotsis
The patient was transferred to a Level 1 center early afternoon on day 6, but developed a low grade fever before departure. Before transfer, she was fatigued but responsive. On arrival to the Level 1 center (2 hr away), she was unresponsive with 103F fever. Blood cultures obtained upon arrival and the patient started on prophylactic cefipime, vancomycin, azithromycin, acyclovir, and voriconazole.
Day 6 at Level 1 center:
    Respiratory PCR panel including influenza negative
    Toxoplasma antibodies negative
    Coccidioides testing negative
    HIV, TB, and mycoplasma testing negative
    Procalcitonin: 0.04
    CT head: continued vasogenic edema right frontalparietal distribution.
    CTA head and neck perfusion: unremarkable
Day 7: another lumbar puncture completed; however, brain biopsy was held in lieu of ID workup.
CSF results from day 7: 172 nucleated cell, 45% neutrophils, 17% lymphocytes, and 38% macrophages.
Glucose 49 and protein 124; lactate 7.7 and LD 54. Blastomyces, toxoplasma, and varicella negative. Histoplasma and CMV negative.
CSF culture negative for growth at 5 days at the Level 1 center.
Day 7: blood culture positive for Gram-positive rods. Added amphotericin to treatment.
Day 8: ampicillin added to antimicrobial treatment.
Day 11: blood cultures positive for *Listeria.*
Day 13: gentamicin added to treatment.
Day 15: MRI imaging showed advancement of disease and comfort care instituted.
The patient expired on day 17.
Brain autopsy: revealed abscesses of the white matter in the frontal, temporal, and occipital lobes as well as the midbrain, pons, and cerebellum with multiple macroscopic leukocytes with perivascular inflammation consisting of lymphocytes and plasma cells consistent with listeriosis.
Diagnosis: *Listeria cerebritis* with bacteremia.

## 2. Discussion

The differential diagnosis of perivascular cerebral edema includes trauma, ischemic injury, cancer, vasculitis, meningitis, or encephalitis.

According to the differential, days 0 and 1 ruled out ischemic injury with negative MRI and CTA head/neck. The history and exam findings were negative for trauma. According to Update.com, “acute bacterial meningitis should be suspected in adults who present with fever and signs of meningeal inflammation. Isolation of a bacterial pathogen from the CSF (by culture or other diagnostic techniques) confirms the diagnosis of bacterial meningitis” [1]. Our patient was afebrile with no signs of nuchal rigidity; thus, antibiotics were not initiated. MRI results and CBC results on admission did not indicate infectious etiology. Within 24 hr of admission, CSF DNA testing for microorganism was negative (the PCR DNA test (BioFire Diagnostics) of the CSF for *Listeria* is 94.2% sensitivity and 99.8% specificity), and she had negative blood and CSF cultures within 2 days, thus providing evidence against the administration of antibiotics. In addition, the negative CSF cytology by day 2 with negative imaging suggestive of malignancy almost ruled out cancer.

An EEG may have provided additional evidence for encephalitis, and a timely brain biopsy may have identified microabscesses. However, with the radiologist differential, collected information, and clinical presentation, we did not treat with antimicrobial therapy.

*Listeria monocytogenes* cerebritis and neuroimaging has been described in the past. According to Haykal et al., in 2 of 3 cases of early *Listeria*, imaging “demonstrated ill defined superficial area of low attenuation with curvilinear gyral enhancement. Although these findings are nonspecific, their early recognition in the proper clinic setting may help institute early antibiotic therapy [2].” However, in the article by Aladro the description of *Listeria* cerebritis in 3 case studies consisted of deep white matter lesions with nodular and ring enhancement [3]. This radiologic description does not coincide with our particular case study. After reviewing the article by Eckburg in which 39 cases of *Listeria* brain abscess lesions were compared, 86% of 33 patients had concomitant bacteremia. The bacteremia is considered an unusual finding in those with brain abscesses caused by bacteria, thus supporting the idea of hematogenous spread as the etiology. Of those 39 cases, 72% of the patients had a fever on presentation; however, they all had abnormal neurologic findings. The CSF culture was positive in a mere 38% of 29 patients. Interestingly brain tissue culture obtained in 19 patients demonstrated positive *Listeria* culture in 18 of those patients (95%), and the remaining patients were s/p ampicillin tx which may explain negative culture. The CNS may be the only site of *Listeria* involvement, and the diagnosis should be considered in any patient who presents with focal neurology signs, mental status changes, and *Listeria monocytogenes* bacteremia [4]. Successful treatment often requires the early involvement of a neurosurgical team in addition to antilisterial antibiiotics. According to Lorber, in patients suspected to have contracted *Listeria*, if CSF fluid and blood cultures are negative, stereotactic biopsy is needed [5].

Mortality rate was higher in those with neurolisterosis and bacteremia (OR 3.67) and was higher in those treated with dexamethasone (OR 4.58). The most frequent comorbidity in those with neurolisterois or bacteremia was solid organ cancer and diabetes [6].

tIn our defense, the study by Brower evaluating 30 cases of *Listeria*, 90% of the patients had fever, 88% had HA, 73% had neck stiffness, and 37% had focal neurologic abnormal exam findings [7]. Our patient was afebrile until day 6 when she was transferred. Similarly in the study by Charlier which included 427 patients with bacteremia, 87% had a fever, and 97% of those with neurolisteriosis were febrile. Blood cultures were positive in 63% of those with neurolisteriosis. CSF culture was positive in 84% of those with neurologic presentation. PCR CSF testing was positive in 63% of those with neurologic presentation (only 16 cases had PCR testing) [6]. Blood cultures for our patient were positive by the third set (first set at the Level 1 center) that identified *Listeria* bacteremia. CSF cultures obtained at 2 different facilities were negative for growth. Only mild pleocytosis was evident in initial CSF analysis. The most recent study by Charlier suggested “neurolisteriosis presents as a combination of neuroradiological images, none of them being specific”: meningeal enhancement 35%, abscess or nodular images indicative of abscess 14%, hemorrhages 15%, contrast-enhancing ventricles or hydrocephalus 10%, white matter images 59%, dilated Virchow-Robin spaces 31%, and brainstem involvement 10% [8].

High index of suspicion for infectious etiology is needed to diagnose CNS listeriosis. In the study by Mylonakis, 29% of patients with *Listeria* meningitis had no meningeal signs [9]. In the study by Charlier, 67% of those with neurolisteriosis had nuchal rigidity [6]. The presentation of cerebritis can have a vasculitic appearance. The vasculitis is frequently multifocal with perivascular necrosis, and the formation of multiple small perivascular abscesses is not amenable to surgical therapy [10].

Worldwide *Listeria* is the most common cause of bacterial meningitis in patients with lymphoma, organ transplant recipient, and those receiving corticosteroid immunosuppression for any reason. The most common risk factors for developing *Listeria* meningitis include malignancy (solid tumor and hematologic; 24%), transplantation (21%), alcoholism/liver disease (13%), immunosuppression/steroid treatment (11%), diabetes mellitus (8%), and HIV/AIDS (7%) [5].

Historically *Listeria* demonstrates bimodal distribution affecting mainly young and older patients, but immune-compromised patients are also at risk. Given the increased prevalence of patients taking immunosuppression for hematologic malignancy therapy, transplant protection, or autoimmune related issues, *Listeria* should be considered when faced with nonspecific, abnormal CNS imaging with no lesions.

In a murine model, TNF found to play a crucial role in the intracerebral control of *Listeria* [11, 12].

Based on review of Revlimid via UpToDate, there are several mechanisms of action. One of the mechanisms of Revlimid is TNF inhibition.

*Revlimid (lenalidomide)*: mechanism of action is angiogenesis inhibitor; antineoplastic agent; and immunomodulator. It selectively inhibits secretion of proinflammatory cytokines (a potent inhibitor of tumor necrosis factor-alpha secretion); enhances cell-mediated immunity by stimulating proliferation of anti-CD3 stimulated T cells (resulting in increased IL-2 and interferon gamma secretion); and inhibits trophic signals to angiogenic factors in cells [13].

Patients with hematologic malignancy are at increased risk for *Listeria*, as mentioned above. In addition, patients taking lenalidomide are at increased risk of serious infection. In the meta-analysis study by Ying et al., the overall incidence of high grade infections was 14.32% (95% CI: 12.08–16.9%) and high grade infection's pooled RR was 2.23 (95% CI: 1.71–2.91, *P* < 0.0001) for all 11 studies (3210 subjects) evaluated [14].

## 3. Conclusion

- In a person with MRI brain imaging that demonstrates cerebral edema who presents with focal neurologic deficits but lacks specific CSF analysis and negative blood cultures, advocate for expedient biopsy (in-house or transfer the patient to capable facility) to quickly determine approach to appropriate treatment. Begin prophylactic antibiotics to include coverage of *Listeria* (such as ampicillin ± gentamicin) until a definitive diagnosis is determined. This is especially true of patients with DM, hematologic malignancy, amyloidosis, and cirrhosis and renal transplant patients.
- Heavily weigh risk and benefits of administering dexamethasone in patients with cerebral edema of undetermined etiology. Consider joint administration of prophylactic antibiotic if neurologic symptoms are intensifying and dexamethasone is instituted.
- Given the increasing prevalence of persons treated with immunosuppressants, specifically TNF inhibitors and steroids, *Listeria* needs to be of high priority on the differential of patients with signs/symptoms of CNS involvement, even with inconclusive CSF analysis and negative blood cultures.
